# How can MCDA tools improve priority setting? Four critical questions

**DOI:** 10.1186/s12962-018-0119-6

**Published:** 2018-11-09

**Authors:** Ole F. Norheim

**Affiliations:** 1 Department of Global Public Health and Primary Care, University of Bergen, Bergen, Norway; 2 Department of Global Health and Population, Harvard T.H. Chan School of Public Health, Boston, MA USA

**Keywords:** Priority setting, Multi-criteria decision analysis, Cost-effectiveness, Equity

## Abstract

This comment argues that four critical questions needs to be resolved before MCDA tools can improve priority setting in health: how to merge the quantitative and deliberative elements of MCDA; how to select criteria; how to weigh them, and whom to bring to the table.

## Background

Baltussen and others have proposed that multi-criteria decision analysis (MCDA) can improve priority setting processes [1–3]. Other approaches include cost-effectiveness analysis [4, 5], criteria-based systems that include cost-effectiveness and other concerns [6–9], programme budgeting and marginal analysis (PBMA) [10], procedural frameworks such as accountability for reasonableness [11], and rights-based approaches [12].

For example, the World Health Organization and the World Bank have championed cost-effectiveness as a key criterion for global and national priority setting [4, 5]. In UK, the National Institute for Health and Care Excellence (NICE) identifies the most cost-effective services through health technology assessment, with input on key values from the Citizen’s Council. In Thailand, the Health Intervention and Technology Assessment Program (HITAP) appraises a wide range of health technologies and public health programs by six criteria: size of population affected, severity of disease, effectiveness of health intervention, variation in practice, economic impact on household expenditure, and equity and social implications [8, 9].

Common for all priority setting approaches is that they seek to provide a ranking of services delivered at different levels of the health system by linking evidence on needs and outcomes with values, principles, and criteria that have support in the population. The approaches differ along at least two axes: (1) technical versus deliberative, and (2) narrow focus versus comprehensive.

MCDA as discussed in this issue of the journal have moved from a more technical framework with a narrow focus towards a deliberative framework allowing for discussion and use of a wide range of criteria and concerns [3]. In my view, MCDA has moved too far from its origin. In the further development of MCDA tools, four critical questions therefore need further scrutiny.

## Technical or deliberative approaches?

Already in 1998, the ethicist Soren Holm said “goodbye to the simple solutions”, arguing that the false dichotomy between substantive and deliberative approaches to priority setting would be replaced by a third phase of integration [6]. A similar development may be seen for MCDA. If both quantification and deliberation is key, how can the two approaches be integrated? Enthusiasm for deliberation should not lead to the abandonment of quantification.

## How many criteria can a decision-maker handle?

The trend of embracing all possible criteria for priority-setting analysis is, in my view, unfortunate. While moving beyond cost-effectiveness as a single criterion is appropriate, quantitative models may not be able to handle an unlimited number of additional concerns without ending up as another black-box approach. Likewise, stakeholders discussing priority setting decisions may not be able to handle and balance 10–15 different considerations for a large set of interventions or services at the same time. Handling such large amount of data is simply not possible in a comprehensive way. The field needs to set priorities among the criteria included in the analytical framework. I am prepared to argue that, at the most general level, we need only four criteria: level and distribution of health outcomes; level and distribution of non-health outcomes.

## Quantitative approaches: additive or multiplicative weights?

Current practice in MCDA for priority setting is to ask key stakeholders which criteria they find relevant and ask them (through discrete choice experiments) to assign weights to each criterion. The MCDA analyst then rank services based on stakeholder values. Often additive weights are used; and sometimes 10–15 criteria are identified in this way. If, say cost-effectiveness is assigned a weight of “20%” though this process (and the remaining 80% is distributed to the other criteria), the result strikes me as problematic. What does “20%” mean? I believe the use of additive weights is at risk of forcing key stakeholders to end up with a rank-order that is different from what they would end up with simply through individual judgment or a purely deliberative process. One possible explanation is the choice of weighing scheme.

Consider this example (Table 1). Three interventions have the same cost, different benefits, and target groups with different incomes. Let us assume that stakeholders care about only two concerns: equity and benefit maximization.

**Table 1:** Three interventions (a–c) with the same cost, different benefits, and target groups with different incomes (PW poverty-weighted)

	A. Multiplicative weights					B. Additive weights				
	Income	Weight	Benefit	PW benefit	PW rank	Income	Rank	Benefit	Rank	Weighted rank
a.	Poor	3	10	30	1	Poor	1	10	3	2
b.	Average	2	14	28	2	Average	2	14	2	2
c.	Rich	1	18	18	3	Rich	3	18	1	2

In A, stakeholders think that benefits to the poor have three times the value of benefits to the rich; in B, stakeholders also think that benefits to the poor are more important than benefits to the rich, and they assign equal weight to ‘equity’ (50%) and ‘benefit’ (50%).

As can be seen from the table, using multiplicative weights would clearly rank the three interventions, while using additive weights would not—in this example. The reason is that additive weights, as used in many forms of MCDA, are applied to ordinal ranks. There is loss of information compared to the multiplicative weighing scheme. Methods for assigning weights to different concerns and their rationale need to be further discussed and explored in MCDA.

## Deliberative approaches: who are invited to the table?

For deliberative approaches to MCDA, issues related to representation and power asymmetries require further clarification [13]. The outcome of MCDA depends crucially on who the participants are. Current practice tends to favor health policy decision-makers and health planners, with some patient or user-participation. Given the technical nature of priority setting, there may be feasibility constraints on who can participate. Yet, this foundational issue for approaches that aim to be more democratic is insufficiently explored. Who is invited to the table matters.

## Conclusion

In summary, recent developments of MCDA tools for priority setting are detached from their theoretical foundations: deliberation without proper quantification can mislead; choosing too may criteria can confuse; using additive weights causes loss of information, and the choice of participants is arbitrary.

## References

[1] Baltussen R (2017). Value assessment frameworks for HTA agencies: the organization of evidence-informed deliberative processes. Value Health.

[2] Marsh K (2016). Multiple criteria decision analysis for health care decision making-emerging good practices: report 2 of the ISPOR MCDA Emerging Good Practices Task Force. Value Health.

[3] Goetghebeur M (2017). The art of priority setting. Lancet.

[4] World Bank, Investing in Health (1993). World development report 1993.

[5] WHO. CHOosing interventions that are cost effective (WHO-CHOICE). 2012. http://www.who.int/choice/en/. Accessed 15 Aug 2012.

[6] Norheim OF (2014). Guidance on priority setting in health care (GPS-Health): the inclusion of equity criteria not captured by cost-effectiveness analysis. Cost Eff Resour Alloc.

[7] World Health Organization (2014). Making fair choices on the path to universal health coverage.

[8] Glassman A, Chalkidou K (2012). Priority-setting in health: building institutions for smarter public spending.

[9] Culyer AJ, Podhisita C, Santatiwongchai B (2016). A star in the east. A short history of HITAP.

[10] Mitton C, Donaldson C (2004). The priority setting toolkit: a practical guide to the use of economics in health care decision making.

[11] Daniels N, Sabin JE (2008). Setting limits fairly: learning to share resources for health.

[12] Yamin A, Gloppen S (2011). Health rights in comparative perspective.

[13] Baerøe K (2009). Priority-setting in healthcare: a framework for reasonable clinical judgements. J Med Ethics.

